# Tetra­aqua­hexa­kis(μ_2_-quinoline-4-carboxyl­ato)diyttrium(III) dihydrate

**DOI:** 10.1107/S1600536808039421

**Published:** 2008-11-29

**Authors:** Chao-Yan Zhang, Qian Gao, Yue Cui, Ya-Bo Xie

**Affiliations:** a College of Environmental and Energy Engineering, Beijing University of Technology, Beijing 100022, People’s Republic of China

## Abstract

In the title centrosymmetric binuclear complex, [Y_2_(C_10_H_6_NO_2_)_6_(H_2_O)_4_]·2H_2_O, each Y^III^ atom is nine-coordin­ated by nine O atoms from five ligands and two water mol­ecules in a slightly distorted monocapped square-anti­prismatic coordination environment. The Y^III^ atoms are separated by a distance of 4.0363 (9) Å. The ligands coordinate in three different modes: chelating, bridging and a mixed chelating bridging mode. In the crystal structure, the binuclear complexes are linked by O—H⋯O and O—H⋯N hydrogen bonds, forming a three-dimensional network.

## Related literature

For transition metal complexes of 4-quinoline­carboxylic acid, see: Bu *et al.* (2005 ▶); Chen *et al.* (2002 ▶); Morsy & Vratislav (2006 ▶).
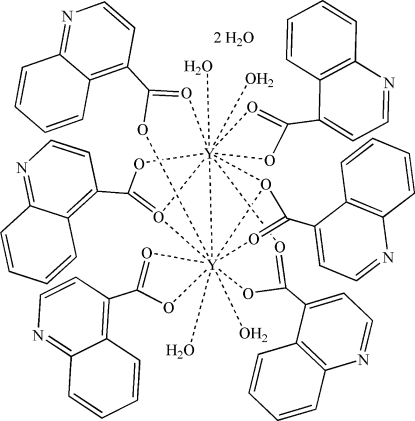


## Experimental

### 

#### Crystal data

[Y_2_(C_10_H_6_NO_2_)_6_(H_2_O)_4_]·2H_2_O
*M* *_r_* = 1318.86
Monoclinic, 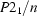

*a* = 11.623 (2) Å
*b* = 16.361 (3) Å
*c* = 15.312 (3) Å
β = 106.03 (3)°
*V* = 2798.7 (10) Å^3^
*Z* = 2
Mo *K*α radiation
μ = 2.15 mm^−1^
*T* = 293 (2) K
0.30 × 0.28 × 0.26 mm

#### Data collection

Bruker SMART CCD area-detector diffractometer
Absorption correction: multi-scan (*SADABS*; Bruker, 1998 ▶) *T*_min_ = 0.565, *T*_max_ = 0.605 (expected range = 0.535–0.573)
9525 measured reflections
4898 independent reflections
3615 reflections with *I* > 2σ(*I*)
*R*_int_ = 0.031

#### Refinement

*R*[*F* ^2^ > 2σ(*F* ^2^)] = 0.034
*wR*(*F* ^2^) = 0.073
*S* = 0.91
4898 reflections
412 parameters
H atoms treated by a mixture of independent and constrained refinement
Δρ_max_ = 0.38 e Å^−3^
Δρ_min_ = −0.30 e Å^−3^

### 

Data collection: *SMART* (Bruker, 1998 ▶); cell refinement: *SAINT* (Bruker, 1998 ▶); data reduction: *SAINT*; program(s) used to solve structure: *SHELXS97* (Sheldrick, 2008 ▶); program(s) used to refine structure: *SHELXL97* (Sheldrick, 2008 ▶); molecular graphics: *SHELXTL* (Sheldrick, 2008 ▶); software used to prepare material for publication: *SHELXTL*.

## Supplementary Material

Crystal structure: contains datablocks global, I. DOI: 10.1107/S1600536808039421/su2081sup1.cif

Structure factors: contains datablocks I. DOI: 10.1107/S1600536808039421/su2081Isup2.hkl

Additional supplementary materials:  crystallographic information; 3D view; checkCIF report

## Figures and Tables

**Table 1:** Selected geometric parameters (Å, °)

Y1—O1	2.398 (2)
Y1—O1*W*	2.337 (3)
Y1—O2	2.461 (2)
Y1—O2*W*	2.370 (3)
Y1—O4	2.3245 (19)
Y1—O5	2.419 (3)
Y1—O6	2.735 (2)
Y1—O3^i^	2.3264 (19)
Y1—O6^i^	2.309 (2)

Symmetry code: (i) 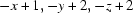.

**Table 2:** Hydrogen-bond geometry (Å, °)

*D*—H⋯*A*	*D*—H	H⋯*A*	*D*⋯*A*	*D*—H⋯*A*
O1*W*—H1*WA*⋯O3*W*^ii^	0.75 (4)	1.99 (4)	2.727 (4)	168 (3)
O2*W*—H2*WA*⋯O3*W*^ii^	0.78 (3)	2.00 (3)	2.751 (4)	161 (3)
O3*W*—H3*WA*⋯N2^iii^	0.87 (4)	1.84 (4)	2.708 (4)	172 (4)
O1*W*—H1*WB*⋯N3^iv^	0.78 (4)	1.96 (4)	2.735 (4)	173 (4)
O2*W*—H2*WB*⋯N1^v^	0.78 (4)	1.99 (4)	2.739 (4)	161 (4)
O3*W*—H3*WB*⋯O2*W*^vi^	0.67 (4)	2.30 (3)	2.865 (4)	143 (3)

Symmetry codes: (ii) 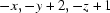; (iii) 
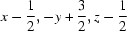
; (iv) 
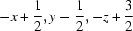
; (v) 
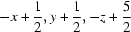
; (vi) 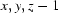.

**Fig. 1.**
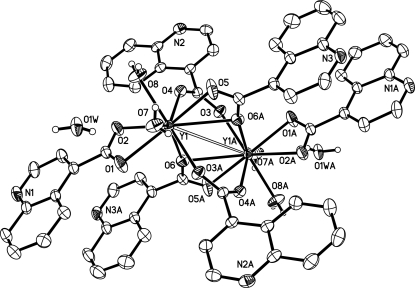
The molecular structure of complex (I), with displacement ellipsoids drawn at the 30% probability level (C-bound H-atoms have been removed for clarity; Symmetry code: (A) -x + 1, -y + 2, -z + 2])

## supplementary crystallographic information

### Comment

Some crystal structures of transition metal complexes with the ligand 4-quinolinecarboxylic acid (HL) have been published previously, for example, with cadmium(II) (Morsy & Vratislav, 2006; Chen *et al.*, 2002), copper(II), cobalt(II) and manganese(II) (Bu *et al.*, 2005). However, no rare earth metal complexes of HL have been reported to date. Herein, we report on the synthesis and crystal structure of a new binuclear yittrium(III) complex of 4-quinolinecarboxylic acid, (I).

The molecuar structute of title compound (I), a centrosymmetric binuclear complex, is illustrated in Fig. 1. The complex is compossed of two yttrium(III) atoms and six 4-quinolinecarboxylate ligands, along with four coordinated and two uncoordinated water molecules. Each yttrium atom is nine-coordinated, with nine oxygen atoms from five ligands and two water molecules, showing a slightly distorted monocapped square-antiprism coordination environment (Table 1). The Y1—O bond distances vary from 2.309 (2) to 2.735 (2) Å, while the Y1···Y1^i ^separation is 4.0363 (9) Å [Symmetry code: (i) -*x* + 1, -*y* + 2, -*z* + 2]. The six 4-quinolinecarboxylate ligands adopt three different coordination modes; chelating, bridging, and a mixed mode of chelating and bridging (Table 1).

In the crystal structure O—H···O and O—H···N hydrogen bonds link the binuclear complexes and uncoordinated water molecules to form a three-dimensional network (Table 2).

### Experimental

A mixture of 4-quinolinecarboxylic acid, sodium hydroxide and yttrium nitrate, in the molar ratio 3:6:1, were dissolved in a mixture of ethanol and water. The resulting solution was filtered and the filtrate allowed to stand in the air for several days. Finally colorless block-like crystals, suitable for X-ray analysis, were obtained with a yield of 25%.

### Refinement

The water H atoms were located in difference Fourier maps and freely refined; O—H = 0.67 (3) - 0.88 (4) Å, with *U*_iso_(H) = 1.5*U*_eq_(O). The C-bound H atoms were included in calculated positions and treated as riding atoms, with C—H = 0.93 Å and *U*_iso_(H) = 1.2*U*_eq_(C).

### Crystal data

**Table:**

[Y_2_(C_10_H_6_NO_2_)_6_(H_2_O)_4_]·2H_2_O	*F*_000_ = 1344
*M**_r_* = 1318.86	*D*_x_ = 1.565 Mg m^−^^3^
Monoclinic, *P*2_1_/*n*	Mo *K*α radiation λ = 0.71073 Å
Hall symbol: -P 2yn	Cell parameters from 6629 reflections
*a* = 11.623 (2) Å	θ = 3.0–27.5º
*b* = 16.361 (3) Å	µ = 2.15 mm^−^^1^
*c* = 15.312 (3) Å	*T* = 293 (2) K
β = 106.03 (3)º	Block, colorless
*V* = 2798.7 (10) Å^3^	0.30 × 0.28 × 0.26 mm
*Z* = 2	

### Data collection

**Table:**

Bruker SMART CCD area-detector diffractometer	4898 independent reflections
Radiation source: fine-focus sealed tube	3615 reflections with *I* > 2σ(*I*)
Monochromator: graphite	*R*_int_ = 0.031
*T* = 293(2) K	θ_max_ = 25.0º
φ and ω scans	θ_min_ = 3.0º
Absorption correction: multi-scan(SADABS; Bruker, 1998)	*h* = −13→13
*T*_min_ = 0.565, *T*_max_ = 0.605	*k* = −19→19
9525 measured reflections	*l* = −18→18

### Refinement

**Table:**

Refinement on *F*^2^	Secondary atom site location: difference Fourier map
Least-squares matrix: full	Hydrogen site location: inferred from neighbouring sites
*R*[*F*^2^ > 2σ(*F*^2^)] = 0.034	H atoms treated by a mixture of independent and constrained refinement
*wR*(*F*^2^) = 0.073	*w* = 1/[σ^2^(*F*_o_^2^) + (0.0401*P*)^2^] where *P* = (*F*_o_^2^ + 2*F*_c_^2^)/3
*S* = 0.91	(Δ/σ)_max_ = 0.003
4898 reflections	Δρ_max_ = 0.38 e Å^−^^3^
412 parameters	Δρ_min_ = −0.30 e Å^−^^3^
Primary atom site location: structure-invariant direct methods	Extinction correction: none

### Special details

**Table:**

Geometry. Bond distances, angles *etc*. have been calculated using the rounded fractional coordinates. All su's are estimated from the variances of the (full) variance-covariance matrix. The cell e.s.d.'s are taken into account in the estimation of distances, angles and torsion angles
Refinement. Refinement of *F*^2^ against ALL reflections. The weighted *R*-factor *wR* and goodness of fit *S* are based on *F*^2^, conventional *R*-factors *R* are based on *F*, with *F* set to zero for negative *F*^2^. The threshold expression of *F*^2^ > σ(*F*^2^) is used only for calculating *R*-factors(gt) *etc*. and is not relevant to the choice of reflections for refinement. *R*-factors based on *F*^2^ are statistically about twice as large as those based on *F*, and *R*- factors based on ALL data will be even larger.

### Fractional atomic coordinates and isotropic or equivalent isotropic displacement parameters (Å^2^)

**Table:**

	*x*	*y*	*z*	*U*_iso_*/*U*_eq_	
Y1	0.33612 (2)	0.97169 (1)	1.01215 (2)	0.0277 (1)	
O1	0.3073 (2)	0.93621 (12)	1.15662 (15)	0.0601 (9)	
O1W	0.1551 (2)	0.91430 (16)	0.92842 (18)	0.0555 (9)	
O2	0.32722 (19)	0.83332 (12)	1.07294 (14)	0.0534 (8)	
O2W	0.1754 (2)	1.05108 (15)	1.0333 (2)	0.0506 (9)	
O3	0.58136 (16)	0.91289 (11)	0.90536 (13)	0.0421 (7)	
O4	0.39176 (15)	0.88949 (10)	0.90683 (13)	0.0382 (6)	
O5	0.27258 (18)	1.06412 (15)	0.88454 (17)	0.0733 (10)	
O6	0.46548 (16)	1.05938 (11)	0.92006 (13)	0.0427 (7)	
N1	0.3198 (2)	0.68024 (15)	1.35199 (18)	0.0506 (10)	
N2	0.4858 (2)	0.66536 (15)	0.71325 (17)	0.0456 (9)	
N3	0.3578 (2)	1.28030 (15)	0.68068 (18)	0.0477 (9)	
C1	0.3158 (2)	0.86114 (17)	1.1454 (2)	0.0372 (10)	
C2	0.3135 (2)	0.80178 (16)	1.22069 (19)	0.0373 (10)	
C3	0.3322 (3)	0.72121 (17)	1.2052 (2)	0.0470 (11)	
C4	0.3354 (3)	0.66315 (19)	1.2734 (2)	0.0549 (13)	
C5	0.2994 (2)	0.75971 (18)	1.3694 (2)	0.0426 (10)	
C6	0.2815 (3)	0.7777 (2)	1.4548 (2)	0.0567 (12)	
C7	0.2608 (3)	0.8547 (2)	1.4768 (2)	0.0644 (14)	
C8	0.2557 (3)	0.9187 (2)	1.4147 (2)	0.0566 (12)	
C9	0.2729 (3)	0.90404 (18)	1.3318 (2)	0.0466 (11)	
C10	0.2956 (2)	0.82397 (17)	1.30585 (19)	0.0371 (9)	
C11	0.4845 (2)	0.87364 (15)	0.88500 (18)	0.0336 (9)	
C12	0.4823 (2)	0.79992 (15)	0.82552 (19)	0.0338 (9)	
C13	0.5075 (2)	0.80853 (17)	0.7444 (2)	0.0420 (10)	
C14	0.5075 (3)	0.73995 (18)	0.6898 (2)	0.0471 (11)	
C15	0.4610 (2)	0.65485 (16)	0.7944 (2)	0.0390 (10)	
C16	0.4388 (3)	0.57510 (18)	0.8204 (2)	0.0537 (13)	
C17	0.4165 (3)	0.56137 (19)	0.9005 (3)	0.0602 (13)	
C18	0.4145 (3)	0.62605 (18)	0.9599 (2)	0.0553 (11)	
C19	0.4325 (2)	0.70432 (17)	0.9368 (2)	0.0443 (11)	
C20	0.4567 (2)	0.72081 (16)	0.85339 (19)	0.0356 (9)	
C21	0.3686 (3)	1.08793 (16)	0.87468 (19)	0.0373 (10)	
C22	0.3669 (2)	1.15372 (16)	0.80556 (19)	0.0343 (9)	
C23	0.3456 (3)	1.23251 (17)	0.8256 (2)	0.0470 (11)	
C24	0.3423 (3)	1.29366 (18)	0.7608 (2)	0.0528 (11)	
C25	0.3782 (2)	1.20227 (18)	0.6586 (2)	0.0402 (10)	
C26	0.3917 (3)	1.1866 (2)	0.5712 (2)	0.0581 (14)	
C27	0.4122 (3)	1.1097 (2)	0.5471 (2)	0.0669 (14)	
C28	0.4202 (3)	1.0448 (2)	0.6076 (2)	0.0633 (12)	
C29	0.4065 (3)	1.05687 (18)	0.6915 (2)	0.0490 (11)	
C30	0.3841 (2)	1.13619 (16)	0.71960 (19)	0.0371 (9)	
O3W	0.0170 (2)	0.96786 (16)	0.11733 (18)	0.0562 (9)	
H1WA	0.102 (3)	0.942 (2)	0.911 (3)	0.078 (15)*	
H2WA	0.122 (3)	1.057 (2)	0.990 (2)	0.061 (13)*	
H3A	0.34270	0.70490	1.14970	0.0560*	
H1WB	0.153 (3)	0.874 (2)	0.901 (3)	0.082 (14)*	
H4A	0.34970	0.60890	1.26140	0.0660*	
H2WB	0.187 (3)	1.092 (2)	1.060 (3)	0.088 (16)*	
H6A	0.28410	0.73580	1.49630	0.0680*	
H7A	0.24980	0.86570	1.53350	0.0770*	
H8A	0.24040	0.97160	1.43040	0.0680*	
H9A	0.26970	0.94720	1.29170	0.0560*	
H13A	0.52480	0.85990	0.72520	0.0500*	
H14A	0.52380	0.74740	0.63420	0.0560*	
H16A	0.43960	0.53150	0.78150	0.0640*	
H17A	0.40220	0.50840	0.91680	0.0720*	
H18A	0.40080	0.61540	1.01580	0.0660*	
H19A	0.42880	0.74690	0.97620	0.0530*	
H23A	0.33340	1.24550	0.88150	0.0560*	
H24A	0.32830	1.34710	0.77580	0.0630*	
H26A	0.38650	1.22940	0.53030	0.0700*	
H27A	0.42100	1.09990	0.48940	0.0800*	
H28A	0.43530	0.99240	0.59000	0.0760*	
H29A	0.41170	1.01290	0.73090	0.0590*	
H3WA	0.013 (4)	0.923 (2)	0.147 (3)	0.117 (17)*	
H3WB	0.072 (3)	0.974 (2)	0.112 (2)	0.055 (13)*	

### Atomic displacement parameters (Å^2^)

**Table:**

	*U*^11^	*U*^22^	*U*^33^	*U*^12^	*U*^13^	*U*^23^
Y1	0.0339 (1)	0.0235 (1)	0.0277 (1)	−0.0005 (1)	0.0121 (1)	0.0002 (1)
O1	0.1087 (18)	0.0350 (12)	0.0497 (14)	0.0074 (12)	0.0438 (14)	0.0098 (10)
O1W	0.0402 (13)	0.0437 (15)	0.0760 (18)	0.0043 (12)	0.0051 (12)	−0.0294 (14)
O2	0.0862 (16)	0.0393 (12)	0.0402 (13)	−0.0050 (11)	0.0266 (12)	0.0044 (10)
O2W	0.0427 (13)	0.0510 (15)	0.0561 (17)	0.0042 (12)	0.0101 (12)	−0.0255 (13)
O3	0.0395 (11)	0.0357 (11)	0.0551 (13)	−0.0049 (9)	0.0196 (10)	−0.0150 (10)
O4	0.0369 (10)	0.0381 (11)	0.0440 (12)	−0.0005 (9)	0.0186 (9)	−0.0099 (9)
O5	0.0437 (13)	0.0940 (18)	0.0874 (19)	0.0106 (12)	0.0266 (13)	0.0621 (15)
O6	0.0416 (11)	0.0361 (11)	0.0428 (13)	0.0065 (9)	−0.0008 (10)	0.0073 (9)
N1	0.0554 (16)	0.0482 (16)	0.0518 (18)	0.0027 (13)	0.0206 (14)	0.0216 (13)
N2	0.0480 (15)	0.0426 (15)	0.0467 (17)	0.0014 (12)	0.0138 (13)	−0.0152 (12)
N3	0.0466 (15)	0.0436 (16)	0.0516 (18)	0.0016 (12)	0.0113 (13)	0.0206 (13)
C1	0.0385 (16)	0.0398 (17)	0.0346 (18)	−0.0005 (13)	0.0122 (13)	0.0088 (14)
C2	0.0348 (15)	0.0405 (17)	0.0375 (18)	−0.0027 (13)	0.0115 (13)	0.0092 (13)
C3	0.059 (2)	0.0392 (18)	0.048 (2)	0.0031 (15)	0.0235 (16)	0.0075 (15)
C4	0.067 (2)	0.0376 (18)	0.067 (3)	0.0054 (16)	0.0303 (19)	0.0186 (16)
C5	0.0377 (16)	0.0518 (19)	0.0395 (19)	−0.0067 (14)	0.0129 (14)	0.0115 (15)
C6	0.062 (2)	0.071 (2)	0.040 (2)	−0.0121 (18)	0.0190 (17)	0.0155 (17)
C7	0.076 (2)	0.083 (3)	0.041 (2)	−0.013 (2)	0.0276 (19)	−0.0033 (19)
C8	0.063 (2)	0.059 (2)	0.051 (2)	−0.0069 (17)	0.0212 (18)	−0.0075 (17)
C9	0.0524 (18)	0.0486 (19)	0.0389 (19)	−0.0032 (15)	0.0126 (15)	0.0057 (15)
C10	0.0321 (15)	0.0444 (17)	0.0355 (17)	−0.0031 (13)	0.0103 (13)	0.0094 (13)
C11	0.0398 (16)	0.0261 (14)	0.0358 (17)	−0.0004 (12)	0.0122 (13)	−0.0014 (12)
C12	0.0300 (14)	0.0335 (15)	0.0380 (17)	0.0013 (12)	0.0098 (13)	−0.0071 (13)
C13	0.0471 (17)	0.0365 (16)	0.0447 (19)	−0.0017 (13)	0.0166 (15)	−0.0053 (14)
C14	0.0510 (18)	0.054 (2)	0.0373 (18)	0.0011 (15)	0.0139 (15)	−0.0100 (15)
C15	0.0336 (15)	0.0333 (16)	0.048 (2)	0.0001 (12)	0.0077 (14)	−0.0098 (13)
C16	0.055 (2)	0.0370 (18)	0.070 (3)	−0.0015 (15)	0.0186 (18)	−0.0114 (17)
C17	0.061 (2)	0.0358 (18)	0.085 (3)	−0.0042 (16)	0.022 (2)	0.0041 (18)
C18	0.063 (2)	0.0447 (19)	0.065 (2)	0.0022 (16)	0.0291 (19)	0.0097 (17)
C19	0.0488 (18)	0.0429 (18)	0.045 (2)	0.0022 (14)	0.0192 (15)	−0.0037 (14)
C20	0.0294 (14)	0.0349 (16)	0.0412 (18)	0.0008 (12)	0.0075 (13)	−0.0052 (13)
C21	0.0468 (18)	0.0366 (16)	0.0309 (16)	0.0046 (14)	0.0146 (14)	0.0048 (13)
C22	0.0288 (14)	0.0371 (16)	0.0368 (17)	0.0047 (12)	0.0086 (12)	0.0112 (13)
C23	0.0570 (19)	0.0436 (18)	0.0441 (19)	0.0067 (15)	0.0202 (16)	0.0062 (15)
C24	0.060 (2)	0.0336 (17)	0.066 (2)	0.0050 (15)	0.0193 (18)	0.0120 (16)
C25	0.0325 (15)	0.0485 (19)	0.0396 (19)	0.0010 (13)	0.0099 (13)	0.0138 (14)
C26	0.053 (2)	0.083 (3)	0.040 (2)	0.0013 (18)	0.0155 (16)	0.0195 (18)
C27	0.065 (2)	0.097 (3)	0.043 (2)	0.005 (2)	0.0221 (18)	−0.002 (2)
C28	0.069 (2)	0.067 (2)	0.057 (2)	0.0112 (19)	0.0224 (19)	−0.0103 (19)
C29	0.0554 (19)	0.0471 (18)	0.046 (2)	0.0072 (15)	0.0164 (16)	0.0032 (15)
C30	0.0345 (15)	0.0421 (17)	0.0354 (17)	0.0026 (13)	0.0109 (13)	0.0082 (13)
O3W	0.0479 (15)	0.0555 (16)	0.0707 (17)	0.0086 (13)	0.0258 (13)	0.0282 (13)

### Geometric parameters (Å, °)

**Table:**

Y1—Y1^i^	4.0363 (9)	C11—C12	1.507 (4)
Y1—O1	2.398 (2)	C12—C20	1.420 (4)
Y1—O1W	2.337 (3)	C12—C13	1.360 (4)
Y1—O2	2.461 (2)	C13—C14	1.399 (4)
Y1—O2W	2.370 (3)	C15—C20	1.417 (4)
Y1—O4	2.3245 (19)	C15—C16	1.409 (4)
Y1—O5	2.419 (3)	C16—C17	1.341 (5)
Y1—O6	2.735 (2)	C17—C18	1.400 (5)
Y1—O3^i^	2.3264 (19)	C18—C19	1.360 (4)
Y1—O6^i^	2.309 (2)	C19—C20	1.408 (4)
O1—C1	1.248 (3)	C21—C22	1.506 (4)
O2—C1	1.240 (4)	C22—C30	1.414 (4)
O3—C11	1.258 (3)	C22—C23	1.364 (4)
O4—C11	1.241 (3)	C23—C24	1.402 (4)
O5—C21	1.230 (4)	C25—C30	1.418 (4)
O6—C21	1.240 (4)	C25—C26	1.413 (4)
O1W—H1WB	0.78 (4)	C26—C27	1.351 (5)
O1W—H1WA	0.75 (4)	C27—C28	1.395 (4)
O2W—H2WB	0.78 (4)	C28—C29	1.352 (4)
O2W—H2WA	0.78 (3)	C29—C30	1.414 (4)
O3W—H3WB	0.67 (4)	C3—H3A	0.9300
O3W—H3WA	0.87 (4)	C4—H4A	0.9300
N1—C5	1.361 (4)	C6—H6A	0.9300
N1—C4	1.296 (4)	C7—H7A	0.9300
N2—C14	1.316 (4)	C8—H8A	0.9300
N2—C15	1.362 (4)	C9—H9A	0.9300
N3—C24	1.307 (4)	C13—H13A	0.9300
N3—C25	1.358 (4)	C14—H14A	0.9300
C1—C2	1.513 (4)	C16—H16A	0.9300
C2—C3	1.367 (4)	C17—H17A	0.9300
C2—C10	1.423 (4)	C18—H18A	0.9300
C3—C4	1.405 (4)	C19—H19A	0.9300
C5—C10	1.425 (4)	C23—H23A	0.9300
C5—C6	1.411 (4)	C24—H24A	0.9300
C6—C7	1.343 (5)	C26—H26A	0.9300
C7—C8	1.405 (4)	C27—H27A	0.9300
C8—C9	1.360 (4)	C28—H28A	0.9300
C9—C10	1.415 (4)	C29—H29A	0.9300
			
O1—Y1—O1W	94.29 (9)	C6—C7—C8	120.4 (3)
O1—Y1—O2	52.93 (7)	C7—C8—C9	120.7 (3)
O1—Y1—O2W	71.97 (9)	C8—C9—C10	120.8 (3)
O1—Y1—O4	129.52 (7)	C2—C10—C5	116.9 (2)
O1—Y1—O5	143.80 (8)	C2—C10—C9	125.4 (3)
O1—Y1—O6	147.07 (7)	C5—C10—C9	117.7 (3)
O1—Y1—C1	26.54 (8)	O3—C11—C12	115.0 (2)
O1—Y1—O3^i^	80.64 (7)	O4—C11—C12	117.3 (2)
O1—Y1—O6^i^	84.75 (8)	O3—C11—O4	127.7 (2)
O1W—Y1—O2	73.28 (9)	C11—C12—C20	121.2 (2)
O1W—Y1—O2W	70.79 (9)	C11—C12—C13	119.8 (2)
O1W—Y1—O4	76.57 (8)	C13—C12—C20	119.0 (2)
O1W—Y1—O5	77.31 (9)	C12—C13—C14	119.9 (3)
O1W—Y1—O6	117.89 (8)	N2—C14—C13	123.3 (3)
O1W—Y1—C1	83.99 (9)	C16—C15—C20	118.9 (3)
O1W—Y1—O3^i^	142.91 (8)	N2—C15—C20	122.6 (2)
O1W—Y1—O6^i^	142.58 (8)	N2—C15—C16	118.4 (3)
O2—Y1—O2W	109.60 (9)	C15—C16—C17	120.7 (3)
O2—Y1—O4	77.23 (7)	C16—C17—C18	120.7 (3)
O2—Y1—O5	147.69 (8)	C17—C18—C19	120.8 (3)
O2—Y1—O6	139.84 (7)	C18—C19—C20	120.0 (3)
O2—Y1—C1	26.42 (8)	C12—C20—C19	123.9 (3)
O2—Y1—O3^i^	126.54 (7)	C15—C20—C19	118.9 (2)
O2—Y1—O6^i^	76.61 (8)	C12—C20—C15	117.1 (2)
O2W—Y1—O4	142.44 (9)	O6—C21—C22	119.8 (3)
O2W—Y1—O5	72.03 (9)	O5—C21—C22	118.5 (3)
O2W—Y1—O6	110.45 (8)	O5—C21—O6	121.8 (3)
O2W—Y1—C1	91.51 (9)	C21—C22—C30	122.1 (2)
O2W—Y1—O3^i^	72.74 (8)	C23—C22—C30	118.8 (3)
O2W—Y1—O6^i^	141.84 (9)	C21—C22—C23	119.1 (3)
O4—Y1—O5	83.27 (8)	C22—C23—C24	119.1 (3)
O4—Y1—O6	69.39 (7)	N3—C24—C23	124.1 (3)
O4—Y1—C1	103.21 (8)	C26—C25—C30	119.2 (3)
O3^i^—Y1—O4	133.97 (7)	N3—C25—C26	118.6 (3)
O4—Y1—O6^i^	75.52 (7)	N3—C25—C30	122.2 (3)
O5—Y1—O6	49.03 (7)	C25—C26—C27	120.1 (3)
O5—Y1—C1	158.26 (8)	C26—C27—C28	120.8 (3)
O3^i^—Y1—O5	85.37 (8)	C27—C28—C29	121.1 (3)
O5—Y1—O6^i^	123.02 (8)	C28—C29—C30	120.2 (3)
O6—Y1—C1	152.67 (7)	C25—C30—C29	118.6 (3)
O3^i^—Y1—O6	69.59 (7)	C22—C30—C29	123.7 (3)
O6—Y1—O6^i^	74.00 (7)	C22—C30—C25	117.7 (2)
O3^i^—Y1—C1	103.59 (8)	C2—C3—H3A	120.00
O6^i^—Y1—C1	78.69 (8)	C4—C3—H3A	120.00
O3^i^—Y1—O6^i^	73.97 (7)	C3—C4—H4A	118.00
Y1—O1—C1	94.29 (18)	N1—C4—H4A	118.00
Y1—O2—C1	91.54 (17)	C5—C6—H6A	120.00
Y1^i^—O3—C11	139.05 (17)	C7—C6—H6A	120.00
Y1—O4—C11	137.44 (17)	C8—C7—H7A	120.00
Y1—O5—C21	102.25 (19)	C6—C7—H7A	120.00
Y1—O6—C21	86.73 (18)	C9—C8—H8A	120.00
Y1—O6—Y1^i^	106.01 (8)	C7—C8—H8A	120.00
Y1^i^—O6—C21	167.1 (2)	C8—C9—H9A	120.00
H1WA—O1W—H1WB	115 (4)	C10—C9—H9A	120.00
Y1—O1W—H1WB	122 (3)	C12—C13—H13A	120.00
Y1—O1W—H1WA	119 (3)	C14—C13—H13A	120.00
H2WA—O2W—H2WB	109 (4)	C13—C14—H14A	118.00
Y1—O2W—H2WA	115 (2)	N2—C14—H14A	118.00
Y1—O2W—H2WB	121 (3)	C15—C16—H16A	120.00
H3WA—O3W—H3WB	112 (4)	C17—C16—H16A	120.00
C4—N1—C5	117.7 (3)	C18—C17—H17A	120.00
C14—N2—C15	118.1 (3)	C16—C17—H17A	120.00
C24—N3—C25	118.0 (3)	C17—C18—H18A	120.00
Y1—C1—C2	176.23 (18)	C19—C18—H18A	120.00
Y1—C1—O2	62.04 (15)	C20—C19—H19A	120.00
O1—C1—O2	121.1 (3)	C18—C19—H19A	120.00
O2—C1—C2	118.4 (2)	C22—C23—H23A	120.00
O1—C1—C2	120.5 (3)	C24—C23—H23A	120.00
Y1—C1—O1	59.18 (16)	C23—C24—H24A	118.00
C1—C2—C10	124.9 (2)	N3—C24—H24A	118.00
C3—C2—C10	118.4 (3)	C25—C26—H26A	120.00
C1—C2—C3	116.7 (3)	C27—C26—H26A	120.00
C2—C3—C4	119.7 (3)	C28—C27—H27A	120.00
N1—C4—C3	124.2 (3)	C26—C27—H27A	120.00
N1—C5—C10	123.0 (3)	C27—C28—H28A	120.00
N1—C5—C6	117.3 (3)	C29—C28—H28A	119.00
C6—C5—C10	119.6 (3)	C28—C29—H29A	120.00
C5—C6—C7	120.7 (3)	C30—C29—H29A	120.00
			
O1W—Y1—O1—C1	67.34 (18)	Y1—O1—C1—C2	175.6 (2)
O2—Y1—O1—C1	2.06 (16)	Y1—O2—C1—O1	3.7 (3)
O2W—Y1—O1—C1	135.54 (19)	Y1—O2—C1—C2	−175.7 (2)
O4—Y1—O1—C1	−8.8 (2)	Y1^i^—O3—C11—O4	0.8 (5)
O5—Y1—O1—C1	141.65 (17)	Y1^i^—O3—C11—C12	179.45 (18)
O6—Y1—O1—C1	−124.49 (17)	Y1—O4—C11—O3	−15.6 (4)
O3^i^—Y1—O1—C1	−149.69 (18)	Y1—O4—C11—C12	165.79 (17)
O6^i^—Y1—O1—C1	−75.11 (17)	Y1—O5—C21—O6	5.4 (3)
O1—Y1—O2—C1	−2.07 (16)	Y1—O5—C21—C22	−174.5 (2)
O1W—Y1—O2—C1	−111.02 (18)	Y1—O6—C21—O5	−4.6 (3)
O2W—Y1—O2—C1	−49.16 (18)	Y1—O6—C21—C22	175.2 (2)
O4—Y1—O2—C1	169.38 (18)	C5—N1—C4—C3	−0.2 (5)
O5—Y1—O2—C1	−136.32 (17)	C4—N1—C5—C10	−0.6 (4)
O6—Y1—O2—C1	135.30 (16)	C4—N1—C5—C6	179.5 (3)
O3^i^—Y1—O2—C1	33.5 (2)	C14—N2—C15—C20	0.6 (4)
O6^i^—Y1—O2—C1	91.48 (17)	C15—N2—C14—C13	0.7 (5)
O6—Y1^i^—O3—C11	−36.8 (3)	C14—N2—C15—C16	−179.4 (3)
O1^i^—Y1^i^—O3—C11	−124.0 (3)	C24—N3—C25—C30	−0.6 (4)
O1W^i^—Y1^i^—O3—C11	151.3 (2)	C25—N3—C24—C23	0.1 (5)
O2^i^—Y1^i^—O3—C11	−95.9 (3)	C24—N3—C25—C26	178.2 (3)
O2W^i^—Y1^i^—O3—C11	162.1 (3)	O2—C1—C2—C3	4.2 (4)
O4^i^—Y1^i^—O3—C11	13.5 (3)	O1—C1—C2—C10	3.5 (4)
O5^i^—Y1^i^—O3—C11	89.5 (3)	O2—C1—C2—C10	−177.0 (3)
O6^i^—Y1^i^—O3—C11	41.7 (3)	O1—C1—C2—C3	−175.3 (3)
C1^i^—Y1^i^—O3—C11	−110.5 (3)	C10—C2—C3—C4	−1.1 (4)
O1—Y1—O4—C11	−99.1 (3)	C1—C2—C10—C9	2.5 (4)
O1W—Y1—O4—C11	176.5 (3)	C3—C2—C10—C9	−178.7 (3)
O2—Y1—O4—C11	−107.9 (3)	C1—C2—C3—C4	177.8 (3)
O2W—Y1—O4—C11	146.5 (2)	C3—C2—C10—C5	0.4 (4)
O5—Y1—O4—C11	98.0 (2)	C1—C2—C10—C5	−178.4 (2)
O6—Y1—O4—C11	49.4 (2)	C2—C3—C4—N1	1.1 (5)
C1—Y1—O4—C11	−103.1 (2)	N1—C5—C10—C2	0.5 (4)
O3^i^—Y1—O4—C11	21.1 (3)	C10—C5—C6—C7	0.1 (5)
O6^i^—Y1—O4—C11	−28.7 (2)	C6—C5—C10—C9	−0.4 (4)
O1—Y1—O5—C21	131.34 (19)	N1—C5—C10—C9	179.6 (3)
O1W—Y1—O5—C21	−148.9 (2)	C6—C5—C10—C2	−179.6 (3)
O2—Y1—O5—C21	−124.1 (2)	N1—C5—C6—C7	−180.0 (3)
O2W—Y1—O5—C21	137.5 (2)	C5—C6—C7—C8	0.5 (5)
O4—Y1—O5—C21	−71.21 (19)	C6—C7—C8—C9	−0.7 (5)
O6—Y1—O5—C21	−2.74 (16)	C7—C8—C9—C10	0.4 (5)
C1—Y1—O5—C21	179.8 (2)	C8—C9—C10—C2	179.3 (3)
O3^i^—Y1—O5—C21	64.11 (19)	C8—C9—C10—C5	0.2 (5)
O6^i^—Y1—O5—C21	−3.4 (2)	O3—C11—C12—C20	122.7 (3)
O1—Y1—O6—C21	−126.05 (18)	O3—C11—C12—C13	−56.0 (3)
O1W—Y1—O6—C21	40.58 (18)	O4—C11—C12—C13	122.9 (3)
O2—Y1—O6—C21	137.62 (16)	O4—C11—C12—C20	−58.4 (3)
O2W—Y1—O6—C21	−37.89 (18)	C13—C12—C20—C15	1.0 (4)
O4—Y1—O6—C21	101.90 (16)	C11—C12—C13—C14	178.9 (3)
O5—Y1—O6—C21	2.66 (16)	C20—C12—C13—C14	0.2 (4)
C1—Y1—O6—C21	−179.40 (18)	C11—C12—C20—C15	−177.7 (2)
O3^i^—Y1—O6—C21	−99.42 (16)	C11—C12—C20—C19	−0.4 (4)
O6^i^—Y1—O6—C21	−177.89 (17)	C13—C12—C20—C19	178.3 (3)
O1—Y1—O6—Y1^i^	51.84 (15)	C12—C13—C14—N2	−1.1 (5)
O1W—Y1—O6—Y1^i^	−141.53 (9)	N2—C15—C20—C12	−1.4 (4)
O2—Y1—O6—Y1^i^	−44.49 (13)	C16—C15—C20—C12	178.6 (3)
O2W—Y1—O6—Y1^i^	140.00 (9)	N2—C15—C20—C19	−178.9 (2)
O4—Y1—O6—Y1^i^	−80.21 (8)	C20—C15—C16—C17	−1.4 (5)
O5—Y1—O6—Y1^i^	−179.45 (12)	N2—C15—C16—C17	178.6 (3)
C1—Y1—O6—Y1^i^	−1.51 (19)	C16—C15—C20—C19	1.1 (4)
O3^i^—Y1—O6—Y1^i^	78.47 (8)	C15—C16—C17—C18	0.2 (5)
O6^i^—Y1—O6—Y1^i^	0.02 (9)	C16—C17—C18—C19	1.5 (6)
O3—Y1^i^—O6—Y1	72.83 (8)	C17—C18—C19—C20	−1.7 (5)
O1^i^—Y1^i^—O6—Y1	154.58 (8)	C18—C19—C20—C12	−176.9 (3)
O1W^i^—Y1^i^—O6—Y1	−115.21 (13)	C18—C19—C20—C15	0.4 (4)
O2^i^—Y1^i^—O6—Y1	−152.32 (9)	O5—C21—C22—C23	75.9 (4)
O2W^i^—Y1^i^—O6—Y1	102.90 (13)	O5—C21—C22—C30	−102.7 (3)
O4^i^—Y1^i^—O6—Y1	−72.29 (7)	O6—C21—C22—C23	−103.9 (3)
O5^i^—Y1^i^—O6—Y1	−0.49 (11)	O6—C21—C22—C30	77.5 (3)
O6^i^—Y1^i^—O6—Y1	0.00 (6)	C21—C22—C30—C29	−1.3 (4)
C1^i^—Y1^i^—O6—Y1	−179.30 (9)	C23—C22—C30—C25	0.0 (4)
O1W—Y1—C1—O1	−112.28 (18)	C23—C22—C30—C29	−179.9 (3)
O2—Y1—C1—O1	−176.3 (3)	C21—C22—C30—C25	178.6 (3)
O2W—Y1—C1—O1	−41.78 (18)	C21—C22—C23—C24	−179.1 (3)
O4—Y1—C1—O1	173.06 (17)	C30—C22—C23—C24	−0.5 (4)
O5—Y1—C1—O1	−81.7 (3)	C22—C23—C24—N3	0.4 (5)
O6—Y1—C1—O1	102.5 (2)	N3—C25—C26—C27	179.9 (3)
O3^i^—Y1—C1—O1	30.82 (18)	C30—C25—C26—C27	−1.2 (5)
O6^i^—Y1—C1—O1	101.06 (18)	N3—C25—C30—C22	0.5 (4)
O1—Y1—C1—O2	176.3 (3)	N3—C25—C30—C29	−179.6 (3)
O1W—Y1—C1—O2	64.02 (17)	C26—C25—C30—C22	−178.3 (3)
O2W—Y1—C1—O2	134.52 (17)	C26—C25—C30—C29	1.6 (4)
O4—Y1—C1—O2	−10.64 (18)	C25—C26—C27—C28	0.1 (5)
O5—Y1—C1—O2	94.7 (3)	C26—C27—C28—C29	0.7 (5)
O6—Y1—C1—O2	−81.2 (2)	C27—C28—C29—C30	−0.2 (5)
O3^i^—Y1—C1—O2	−152.88 (16)	C28—C29—C30—C22	179.0 (3)
O6^i^—Y1—C1—O2	−82.64 (17)	C28—C29—C30—C25	−0.9 (5)
Y1—O1—C1—O2	−3.8 (3)		

Symmetry codes: (i) −*x*+1, −*y*+2, −*z*+2.

### Hydrogen-bond geometry (Å, °)

**Table:**

*D*—H···*A*	*D*—H	H···*A*	*D*···*A*	*D*—H···*A*
O1W—H1WA···O3W^ii^	0.75 (4)	1.99 (4)	2.727 (4)	168 (3)
O2W—H2WA···O3W^ii^	0.78 (3)	2.00 (3)	2.751 (4)	161 (3)
O3W—H3WA···N2^iii^	0.87 (4)	1.84 (4)	2.708 (4)	172 (4)
O1W—H1WB···N3^iv^	0.78 (4)	1.96 (4)	2.735 (4)	173 (4)
O2W—H2WB···N1^v^	0.78 (4)	1.99 (4)	2.739 (4)	161 (4)
O3W—H3WB···O2W^vi^	0.67 (4)	2.30 (3)	2.865 (4)	143 (3)
C3—H3A···O2	0.93	2.39	2.721 (4)	101
C9—H9A···O1	0.93	2.24	2.868 (4)	125
C19—H19A···O2	0.93	2.56	3.421 (4)	154
C19—H19A···O4	0.93	2.55	3.081 (3)	117

Symmetry codes: (ii) −*x*, −*y*+2, −*z*+1; (iii) *x*−1/2, −*y*+3/2, *z*−1/2; (iv) −*x*+1/2, *y*−1/2, −*z*+3/2; (v) −*x*+1/2, *y*+1/2, −*z*+5/2; (vi) *x*, *y*, *z*−1.
